# Recurrent ovarian mixed germ cell tumor with unusual malignant transformation: a case report

**DOI:** 10.1186/s13048-018-0476-y

**Published:** 2019-01-10

**Authors:** Yi-Le Lee, Chiung-Ru Lai, Ming-Shyen Yen

**Affiliations:** 1 Department of Obstetrics and Gynecology, Taipei Veterans General Hospital and National Yang-Ming University, 201, Section 2, Shih-Pai Road, Taipei, 112 Taiwan, Republic of China; 2 Department of Pathology and Laboratory Medicine, Taipei Veterans General Hospital, Taipei, Taiwan; 3 School of Medicine, National Yang-Ming University, Taipei, Taiwan

**Keywords:** Ovarian mixed germ cell tumor, Chemotherapeutic retroconversion, Teratoma malignant transformation

## Abstract

**Background:**

The value of this report is the identification of late recurrence with an extremely unusual combination of malignant transformation. In particular, the retroconversion of immature to mature teratoma as well as a somatic-type malignant transformation were both observed postchemotherapeutically in our case.

**Case presentation:**

We report the case of a 20-year-old girl who completed fertility-sparing surgery and chemotherapy under the diagnosis of ovarian mixed germ cell tumor (immature teratoma and yolk sac tumor) and experienced subsequent recurrence 4 years after a second debulking surgery with a somatic type malignant transformation (teratoma with melanoma and leiomyosarcoma). Multiple metastases developed after a third debulking surgery, and the patient survived for 18 additional months.

**Conclusions:**

Recurrent disease after repeated cytoreduction and chemotherapy hints a poor outcome despite a generally excellent long-term survival rate among ovarian germ cell malignancies. It is important for clinicians to distinguish those at risk of poorer outcomes and establish individualized postoperative surveillance. Fertility-compromising surgery may be considered in selected patients.

## Background

Malignant germ cell tumors account for less than 5% of all ovarian neoplasms [1]. These lesions mostly occur in the first two decades of life, whereas immature teratomas are the second most frequent type (20%~ 35.6% of all cases) [2, 3]. For pure form tumors or a component of mixed germ cell tumors, the prognosis of immature teratomas is greatly dependent on the initial stage and histological grading, which are determined by the levels of immature neural elements that are considered to be evoluted from a malignant clone [4, 5]. Comprehensive surgical staging is warranted. If fertility is desired, unilateral or bilateral salpingo-oophorectomy can be considered in selected patients, with every effort to confirm the extent of initial disease and achieve maximum cytoreduction. Although optimal fertility-sparing cytoreduction followed by chemotherapy appears to achieve a promising outcome for women of childbearing age, refractory courses occurred in a few cases with possible mortality. A recent combined data analysis reviewing 81 adult ovarian immature teratomas revealed a relapse rate of 0, 3.7, and 20% in cases graded 1, 2, and 3, respectively [6]. Among grade 3 disease, the 5-year event-free survival rate decreased from 91% in stage I/II to 65% in stage III/IV. Those with incomplete resection exhibited the worst outcomes. Most cases of recurrence developed within 2 years after primary therapy [7–11].

When germ cell tumors are comprised of mixed subtypes, an advanced challenge in diagnosis and treatment is noted. Mixed germ cell tumors represent only less than 1% in all ovarian germ cell tumors, in contrast to the testis, in which its proportion is up to 33% [12]. Smith HO analyzed 1262 cases of malignant germ cell tumors and reported a lowest overall survival rate (67.4%, compared with 89.1% in dysgerminomas and 84.0% in immature teratomas) in the group of mixed germ cell tumors during a median follow-up of 126 months [2]. The combination with a yolk sac tumor, which is also known as an endodermal sinus tumor, implies a more aggressive tendency [13]. The current literature suggests a beneficial role of salvage treatment in chemorefractory patients, including patients undergoing repeated surgery and second-line chemotherapy [11, 14]. Prompt awareness of tumor marker elevation and immunochemistry-aided diagnosis were reinforced by recent studies [2, 15, 16].

## Case presentation

A 16-year-old girl was referred to our service from another hospital for further management of recurrent germ cell malignancy. Her initial presentation was abdominal bloating with an adnexal 18-cm mass and tumor marker elevation (Alpha-fetoprotein (AFP): 131.53 (0–20) ng/mL, CA 125: 521 (0–35) U/mL, Lactate dehydrogenase (LDH): 242 (98–192) IU/L). She received optimally debulked fertility-sparing staging surgery (unilateral salpingo-oophorectomy, unilateral pelvic and para-aortic lymph node dissection, omentectomy, and cul-de-sac tumor excision) followed by chemotherapy with 4 cycles of BEP (bleomycin, etoposide, and cisplatin) at age 14. Final pathology reported a mixed germ cell tumor composed of mainly immature teratoma (grade 3, FIGO stage IIIC, pT3cN0M0) with components of embryonal carcinoma (23%) and yolk sac tumor (3%).

During follow-up, disease recurrence was suspected due to tumor marker elevation (AFP: 118.82 ng/mL, CA 125: 58.82 U/mL) and pelvic cystic lesions with ascites on abdominal computed tomography (CT) scan. Thus, she was referred to our hospital and received second fertility-sparing debulking surgery. Intra-operative findings were a 6 × 5-cm solid tumor at omentum, another gray tan soft tissue measuring 5 × 3 cm upon bladder, and some small cul-de-sac tumors. No residual disease was noted. All lesions were reported to be metastatic mixed germ cell tumors, which was mainly composed of an immature teratoma with focal areas of yolk sac tumors. The teratoma component is composed of squamous epithelium, intestinal-type epithelium, respiratory epithelium, mesenchymal tissue and neuroglial tissue. The immature element is found focally and is composed of fetal-type epithelium and mesenchymal tissue. This neuroglial tissue is characterized by GFAP (glial fibrillary acidic protein)-positive astrocytic cells and scattered ganglion cells. Adjuvant chemotherapy with 4 cycles of EP (etoposide and cisplatin) was completed uneventfully.

**Fig. 1:**
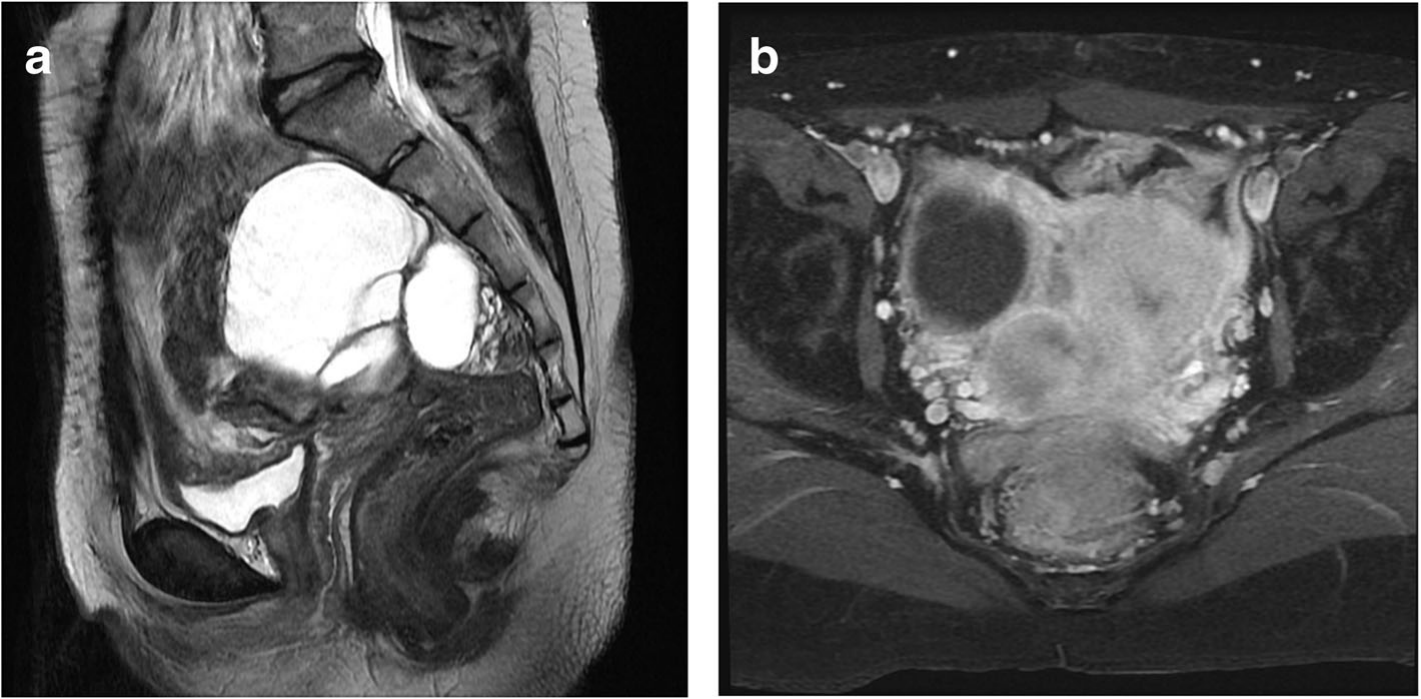
MRI T2-weighted images of pelvic cavity. **a** Sagittal view showing a 7.8 × 7.3-cm cystic mass with internal septa and blood content at right adnexa. **b** Axial view showing some enhancing soft tissue at the cul-de-sac with restricted diffusion on diffusion-weighted imaging, in favor of tumor seedings

**Fig. 2:**
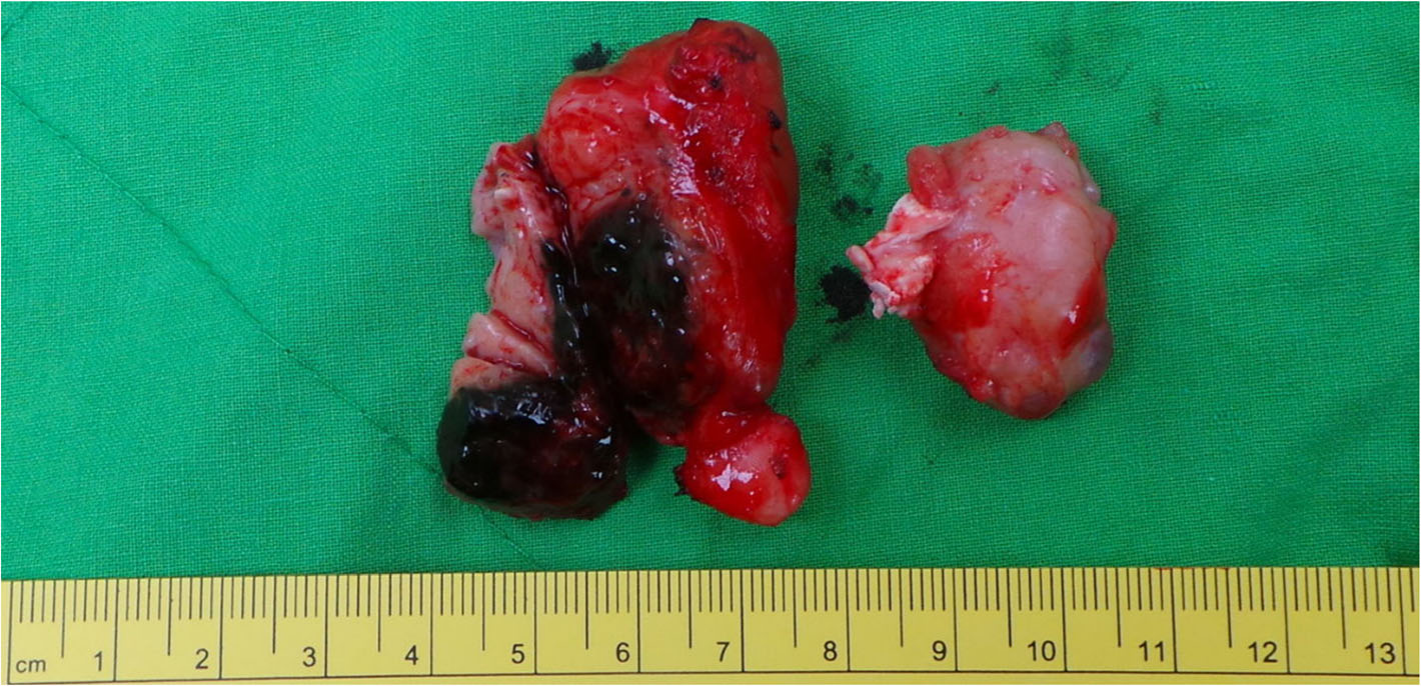
Gross finding of intra-pelvic tissue. Two pieces of tan soft to firm tissue were labeled as tumors on the bladder surface measuring 5 × 3 × 3 cm and 3.2 × 2.5 × 1.6 cm. Dark pigmentation is noted within the 5-cm tissue

**Fig. 3:**
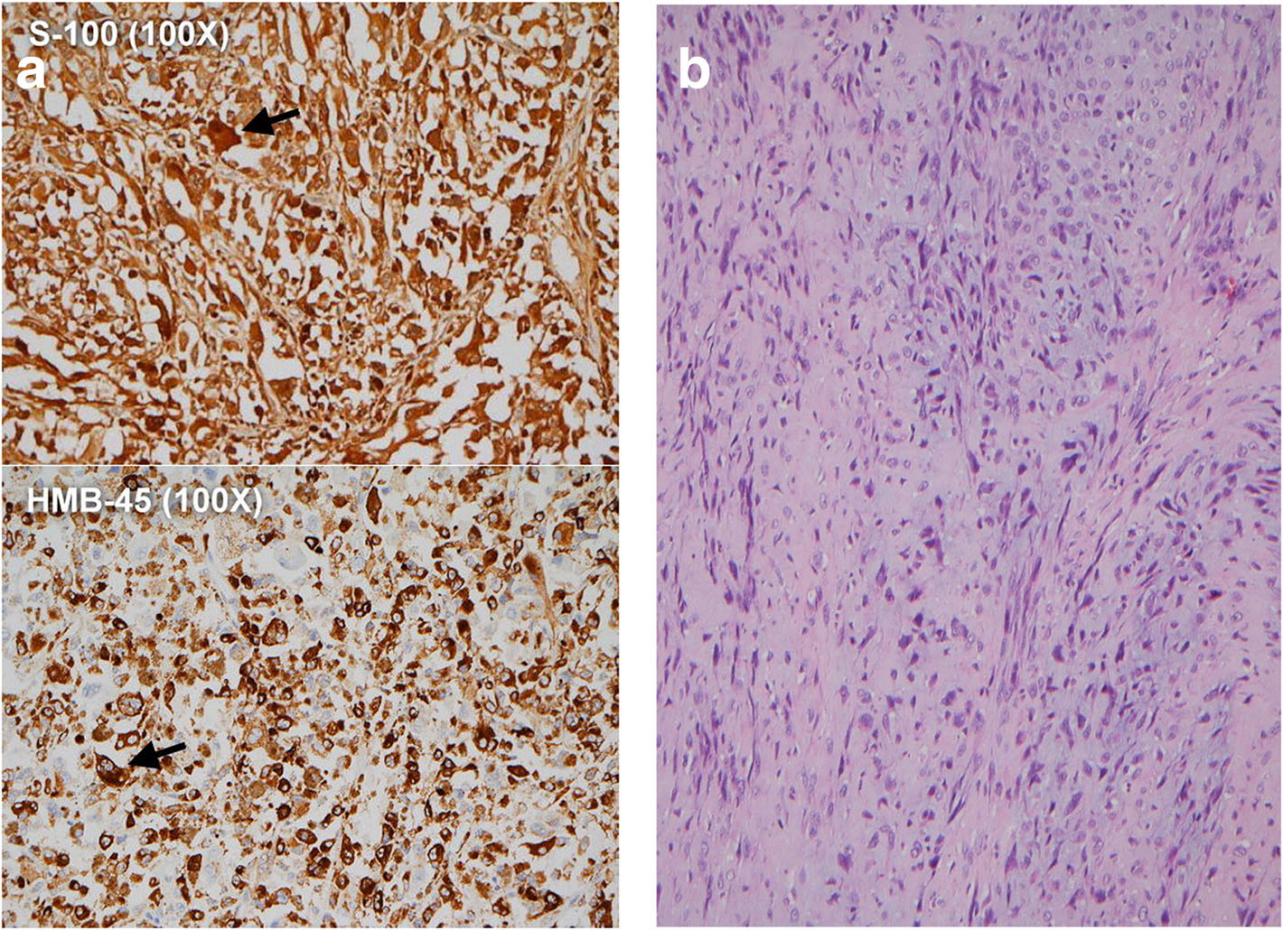
Histological examination of surgical specimen. Sections of the bladder tumor reveal a mixed germ cell tumor that is mainly composed of a teratoma with somatic-type malignancy (melanoma and leiomyosarcoma). No immature component is identified. The yolk sac tumor component is only observed in focal areas. The melanoma is composed of sheets of neoplastic cells with melanin pigment, which is demonstrated with Fontana-Masson stain. The melanoma tumor cells are immuno-reactive for S-100, Melan A and HMB-45. The leiomyosarcoma cells are positive for SMA and HHF-35 but nonreactive for h-Caldesmon. **a** Sheets of melanoma cells with melanin pigment (original magnifications X100). Positive staining for S-100 is noted in both the nuclei and cytoplasm of cells, and the cytoplasm is positive for HMB-45. **b** Hematoxylin and eosin-stained sections (original magnifications X100) reveal spindle-shaped tumor cells with marked nuclear atypia and frequent mitotic figures (> 10/10 hpf) compatible with leiomyosarcoma (positive for SMA and HHF-35)

However, 4 years later (at age 20), the magnetic resonance imaging (MRI) detected an 8 × 7-cm cystic mass at the right adnexa with suspicious pelvic tumor seedings (Fig. 1). AFP was 42.31 ng/mL, and CA 125 was 13.5 U/mL. The patient underwent a third conservative debulking surgery with excision of a right ovarian cystic lesion and pelvic tumors. The surgery was considered optimal, but some residual tumors remained in the cul-de-sac, all of which were less than 10 mm in diameter. The pathology results of the right ovarian tumor suggested a teratoma and associated mucinous cystadenoma, whereas the intrapelvic tissue sections revealed a mixed germ cell tumor composed of teratoma with somatic type malignancy (melanoma and leiomyosarcoma) and foci of yolk sac tumor (Figs. 2 and 3). No immature component is identified within the teratoma. Pelvic recurrence and multiple metastases were noted 8 months later after a third debulking surgery. Biopsy of sacral bone, lung, and liver were compatible with the presence of sarcomatous cells. The patient received palliative radiotherapy, salvage chemotherapy with high-dose doxorubicin and ifosfamide for leiomyosarcoma, and immunotherapy with pembrolizumab for melanoma. She died of disease 10 months later.

## Discussion

It is known that immature teratomas are associated with mature teratomas. Approximately 26% of synchronous typical mature teratomas were detected in cases of immature teratomas, including contralateral ovary (approximately 40% of teratomas) [17]. The concept behind the scene is that ovarian teratomas originate from benign germ cells, whereas the evolution of a malignant clone results in its immature elements, which affect the prognosis most [12]. Occasionally, microfoci of immature tissue (< 21 mm^3^) may present simultaneously in mature teratomas without clinical significance [17]. An improved survival rate via so-called chemotherapeutic retroconversion was recognized among immature teratomas [18, 19]. Through the phenomenon undergoing tissue maturation, a distinct role and favorable outcomes of salvage treatment in immature teratomas are noted compared with other ovarian malignant germ cell tumor types [14, 20]. A similar phenomenon, namely, growing teratoma syndrome, was significantly reported in males with testicular nonseminomatous tumors. Only 101 cases were listed in a literature review of ovarian growing teratoma syndrome with highlights on persistent mass lesions with normalization of serum tumor markers [21, 22]. Either differentiation of malignant cells into mature elements or a chemotherapy effect was thought to be the possible mechanism.

On the other hand, malignant transformation of mature teratoma is uncommon, occurring in 0.19–2% of dermoid cysts in patients often greater than 60 years of ages [23]. These lesions are generally an adult type malignancy in contrast to an immature teratoma that is most commonly a squamous cell carcinoma (80%), and a variety of cell types, including carcinoid, adenocarcinoma, thyroid and basal cell carcinoma, melanoma, and sarcoma, are noted [24]. In particular, a high rate of histopathological aneuploidy and malignant potential were noted in teratoma components that underwent chemotherapeutic retroconversion in the cases of nonseminomatous testicular tumor with secondary malignancy [25–27]. Complete resections often reproduce favoring curative results. Among ovarian dermoid-associated malignancies, a worse prognosis is noted for melanomas (greater than half of deaths occurred within 18 months) [28, 29]. Primary ovarian melanoma is rare with approximately 50 cases to date, [29] and the diagnosis is more convincing with unilaterality, junctional change, and exclusion of another primary site [30]. Various adjuvant chemotherapies and immunotherapies with biologic agents have been used given the poor outcomes that are even worse than cutaneous melanoma [31].

An ongoing debate exists regarding how teratomatous elements develop in mixed germ cell tumors. Current histogenetic analysis hypothesized that the presence of isochromosome 12p (i(12p)) and 12p amplification in mixed germ cell tumors as well as their absence in pure form teratomas (whether mature or immature) supported its derivation from its nonteratomatous components rather than a parthenogenetic origin of a nontransformed germ cell [32].

In the outcome analysis of 20 cases with malignant ovarian germ cell tumors after salvage surgery from Munkarah A et al., [20] a third debulking surgery was reported in only two out of eight immature teratoma patients with greater than 15-year remission in both cases (186 and 202 months respectively). Rezk Y et al. reported a case similar as ours [18] with a grade 3 immature teratoma with foci of yolk sac tumor who failed two different regimens of platinum-based chemotherapy. The patient remained disease-free greater than 4 years after secondary debulking surgery. The value of this report is the identification of late recurrence with an extremely unusual combination of malignant transformation. The disease relapsed 2 years after primary treatment and again 4 years after salvage therapy, which is longer than generally expected. With advanced initial histology grade and stage, the disease exhibited an aggressive behavior with refractory courses and distant metastases despite a second round of chemotherapy and third debulking surgery. Interestingly, the retroconversion of an immature to mature teratoma as well as somatic-type malignant transformation were both observed postchemotherapeutically in our case, which added to the disease complexity.

Post-treatment surveillance in recurrent female germ cell tumors is not yet well-established. Given the sensitivity of serum markers in disease detection and the concern of radiation, the Society of Gynecologic Oncologists recommended that imaging is not indicated before laboratory or clinical findings [33].

Some novel therapies were proposed for platinum-resistant and multiply relapsed germ cell tumors, mainly in male patients [34]. High-dose chemotherapy plus hematopoietic stem cell rescue was potentially effective in progressively growing testicular cancer [35]. Attempts on target agents, such as sunitinib and bevacizumab, were made based on the role of vascular endothelial growth factor in metastatic germ cell tumors [36, 37]. The pathogenesis of germ cell tumors appears different from other somatic tumors with much less involvement of mutations in those common oncogenes or tumor suppressor genes [38]. Whole-genome sequencing may help determine the complicated features of malignant ovarian germ cell tumors provide important information for personalized medicine.

## Conclusions

Given the generally chemo-sensitive feature and reproductive needs of these patients, optimal cytoreduction is warranted to achieve a maximum benefit from subsequent chemotherapy. Although long-term survival rate is excellent among ovarian germ cell malignancies under current treatment strategies, relapsing disease after chemotherapy indicates a poor prognosis especially in cases with higher grades and stages. Post-treatment recurrence may have late presentation despite a stable period after salvage therapy, whereas the retroconversion and somatic-type malignant transformation both existed. To the best of our knowledge, it is the first report of ovarian mixed germ cell tumor with both these two phenomenon observed at the same time. We propose that curative surgery that compromises fertility should be considered in these patients with advanced and relapsing disease. Given the rarity of the disease, it is important for clinicians to distinguish those at risk of poorer outcomes and establish individualized postoperative surveillance before the presence of more valid evidence-based guidance. Novel therapeutic agents are under investigation and expected to deliver promising results.

## Abbreviations

AFP: Alpha-fetoprotein
BEP: bleomycin, etoposide, and cisplatin
CT: computed tomography
EP: etoposide and cisplatin
HCG: human chorionic gonadotropin
MRI: magnetic resonance imaging
